# Examining the Postdictive Validity of Self-Report Big Five Personality Traits with Objective Recordings of Online Behaviors: A Ten-Year Retrospective Study Using Facebook Page Likes

**DOI:** 10.1016/j.heliyon.2024.e32746

**Published:** 2024-06-14

**Authors:** Davide Marengo, Michele Settanni

**Affiliations:** Department of Psychology, University of Turin, Italy

**Keywords:** Personality, Digital traces, Validity, Social media, Longitudinal data

## Abstract

The present study investigated the postdictive validity of self-report Big Five personality traits using over ten years of recording of online behaviors, namely Facebook Page Likes. We explored how personality traits correlate with interests and preferences expressed through Facebook Likes recorded up to ten years before the personality assessment and examined the consistency of these correlations over time. The recruited sample consisted of 601 adult Facebook users, predominantly young adults, with 73.70 % female and 26.30 % male participants. Facebook Page Likes data were analyzed using topic modeling techniques to extract meaningful indicators of individual difference in user interests. Findings revealed significant associations between personality traits and participants' interests as expressed using Likes over ten years of online activity. Conscientiousness showed consistent negative correlations with leisure and entertainment interests. Openness to Experience positively correlated with interests in artistic and cultural fields, including non-profit organizations, theaters, musicians, and entertainment and media. Extraversion demonstrated positive correlations with social entertainment, such as nightclubs and restaurants. Agreeableness and Emotional Stability did not show significant average associations. There was a negative correlation between the number of Likes and Conscientiousness, suggesting that individuals that are more conscientious express fewer Page Likes. Conversely, a positive correlation existed between Page Likes and Openness. Overall, correlations were small but mostly consistent over time, although correlations with the Openness trait suggested a stronger association with more recent interests. This research underscores the enduring influence of personality on online behaviors, including activity on social media.

## 1 Introduction

In the context of personality psychology, Big Five personality traits – namely, Openness to Experience, Conscientiousness, Extraversion, Agreeableness, and Neuroticism (often referred to as its reverse, i.e., Emotional Stability) - have long been considered a robust framework for understanding human personality [1,2]. Historically, the predictive validity of these traits has been explored in various contexts, ranging from academic achievement to workplace performance [3,4]. Of note, robust findings indicate that these traits are expected to become more stable as individuals approach adulthood [[5], [6], [7], [8], [9]]. The stability of the Big Five traits over time also translates into consistent behaviors and patterns of interaction with the environment. For example, highly conscientious persons are more likely to consistently display organized, dependable behaviors across different contexts and stages of life than individuals low on the trait [10]. The advent of the Internet, and particularly the explosion of social media, presents an unprecedented opportunity to examine how these traits relate to behavior in a novel context: their validity in relation to objective recording of online behaviors over an extended period. To our knowledge, no previous study has explored this research area.

In this view, the present study aims to retrospectively investigate the postdictive validity of Big Five personality traits over a decade of online behavior data, namely a dataset of Facebook Page Likes expressed by users up to ten years before the personality assessment. Facebook, with its global reach and extensive user base - over 3 billion monthly active users worldwide [11] - continues to represent an ideal platform for such an analysis [[12], [13], [14]]. Facebook Page Likes are voluntary, public endorsements of pages created on Facebook by other users, companies, institutions, and organizations, either public or private. As such, they offer a unique window into an individual's preferences, interests, and potentially, their underlying personality traits [15]. The ten-year span of this study provides a robust temporal framework, allowing for the examination of how consistently over time Big Five personality traits are linked with online behaviors. This long-term perspective is crucial, as it accounts for the evolution of the online platform, the maturation of individual users, and the potential shifts in behavioral tendencies as individuals navigate different life stages [16]. The decade-long time frame is also consistent with studies on the stability personality over the life span [[6], [7], [8], [9]].

In delving into this retrospective analysis, this paper seeks to contribute to the existing body of knowledge in several ways. First, it aims to enrich our understanding of the relationship between personality traits and online behaviors, with a focus on social media behaviors [17]. Second, we aim to assess the relevance of online behavioral data, particularly preferences for specific content on social media, as reflective of underlying personality constructs [18,19]. A crucial aspect of this investigation is to establish the stability of the observed associations over time. This step lays the groundwork for affirming the enduring influence of personality traits on online behaviors. In short, our research is driven by two primary hypotheses.

1) Hypothesis 1: Big Five personality traits will show theoretically meaningful associations with preferences emerging from Facebook page Likes, reflecting a digital footprint that aligns with their personality characteristics.
2) Hypothesis 2: The associations emerging between personality traits and preferences for specific online content will prove to be stable in strength over the considered time frame.

Note that in the present study, to extract preferences from Facebook Page Likes data we use topic modeling techniques well-regarded for their efficacy in uncovering latent themes from vast collections of unstructured data [20], including Facebook Likes data (e.g., Ref. [21]). This approach allows to model users' interests and preferences as manifested in their Page Likes, transforming digital markers into more coherent topics that reflect underlying content domains.

## 2 Material and methods

### 2.1 Sample and procedure

We sought participants by spreading online a link to a specifically devised web application. This application, which operated through a browser, was specifically created for the study, and underwent the review Facebook process before being activated. Its initial page featured a form to inform participants about the research. Additionally, a Facebook login button was utilized both to gather informed consent and to permit access to participants' Facebook data. Criteria for participation included proficiency in Italian, being of legal age (≥18 years old) and having an active Facebook account. Upon entering the app, participants completed various questionnaires relevant to the study, and their Facebook data was automatically collected. Participants were presented with an online survey including questions regarding demographic variables (gender, age, education level) and a self-report measure providing a brief assessment Big Five traits, namely the Ten Item Personality Inventory (TIPI, [22,23]), and additional psychological measures that are not discussed here. The procedure of the study was reviewed and approved by the ethics committee of the University of Turin, Italy, with the approval number: n° 88721.

To recruit participants, we used a snowball sampling method, initiating with a primary group of 10 university students. Data collection took place from March to June 2018. Eventually, 2998 users accessed the application, while a subsample of 2349 participants provided us with both self-report data and authorization to access to their Facebook Page Likes (589 men, 1760 women; 67 % in the 18–25 age group, and 26 % in the 26–35 age group, 7 % aged >35). Please note that a partial overlap exists between the data examined here and other published studies [[24], [25], [26], [27], [28], [29], [30]]. In the present study, analyses were performed on a subsample of participants who provided us with both self-report data and authorization to access to their Facebook Page Likes; in particular, we selected those with at least 10 years of Facebook Page Likes activity. The resulting sample consists of 601 adult Facebook users. Specifically, 443 participants are female, accounting for 73.70 % of the sample, while the male group consists of 158 individuals representing 26.30 %. When we look at the age distribution, the cohort is predominantly young but slightly older than the initial sample. The largest segment, comprising 45.40 % of the group, falls within the 18–25 age bracket (N = 273), 34.90 % is in the 26–30 (N = 210 participants), 10.50 % are between 31 and 35 (N = 63), and 9.20 % are 36 or older (N = 55). The educational background of participants leans towards higher education. Those with a high school diploma were 33.60 % of the sample (N = 202), 37.80 % participants (N = 227) held a bachelor's degree, and 27.80 % held a master's degree (N = 167). A small fraction, 5 individuals (0.80 %), reported holding a middle school diploma as their highest educational attainment.

### 2.2 Instruments

***Big Five Personality Traits.*** As noted above, individual differences in Big Five traits were assessed using the Italian version of the TIPI questionnaire [22], a brief tool providing scores for the Openness to Experience, Conscientiousness, Agreeableness, Extraversion, and Emotional Stability (Neuroticism reversed). The TIPI is designed for situations where a longer, more detailed personality assessment is not practical due to time constraints or survey length considerations, which may be of specific interest in online studies data [31]. Each of the Big Five dimensions is assessed with two items: one item phrased in a direction indicative of a high trait level and the other in a direction indicative of a low trait level. Respondents rate their agreement with each item on a 7-point scale, ranging from 1 (Disagree strongly) to 7 (Agree strongly). Despite its brevity, the TIPI has been found to have reasonable reliability and validity, making it a useful tool for researchers and practitioners needing quick personality assessments. The TIPI has shown convergent validity with longer assessments, including the BFI-44 [22] and NEO-PI-R [23]. In the Italian version, Cronbach's alpha is expected to range from 0.38 (Agreeableness) to 0.72 (Extraversion), although this metric may not be appropriate for determining reliability since the scales only include 2 items. In turn, test-retest reliability is expected to be quite high, ranging from 0.79 to 0.90, depending on the trait [22]. In the present study, on average we observed the following scores: Extraversion: Mean = 4.01 (SD = 1.58); Agreeableness: Mean = 5.29 (SD = 1.11); Conscientiousness Mean = 5.17 (SD = 1.31); Emotional Stability: Mean = 4.12 (SD = 1.42); Openness: Mean = 5.11 (SD = 1.11).

***Facebook Page Likes.*** Facebook page Likes were collected by submitting a request through the Facebook Graph application-programming interface (API), as is typical when collecting data on Facebook [14]. Features extracted included all expressed Likes to Facebook pages by the user since he/she joined Facebook, including a timestamp indicating when the user liked the page, and the category of the liked page. Because of the sparsity of the page Likes matrix (N = 434252 unique Facebook pages), we coded participants' Likes on pages based on the page category information provided by Facebook. For each Facebook page, the page category provides information about the type of content that users can expect to find on the page (e.g., Depeche Mode's Facebook page is listed under the “Musician/band” category; eBay's page on Facebook is listed as “Retail company”). Thus, for each user, we generated the count of Likes in each page category; eventually, this step resulted in the scoring of each participant based on n = 1231 unique features per 10 distinct years, each representing the total count of Likes expressed by the user to a specific page Likes category during a specific year prior to the personality assessment. Descriptive statistics for collected Facebook Page Likes data that are reported in Table 1.

**Table 1:** Descriptive statistics for Facebook page likes by number of years from personality assessment.

	Descriptives for Total Number of Facebook Page Likes			
Time	Mean	SD	Min	Max
t0	84.53	107.24	2	935
t1	92.60	108.46	1	979
t2	97.64	107.44	2	677
t3	94.13	128.01	1	1568
t4	72.25	104.92	1	1818
t5	45.98	64.71	1	940
t6	58.26	89.15	1	1024
t7	55.64	75.91	1	799
t8	28.49	31.15	1	262
t9	26.06	39.24	1	494

Note. t0 indicate the same year of personality assessment, and t1-t9 indicate 1–9 years prior to the original personality assessment (t0).

### 2.3 Data analysis

To extract meaningful topics reflecting users’ online interests, we applied a data mining approach to Facebook Page Likes categories counts. Topics of interest were extracted from the data applying a topic model analysis using Latent Dirichlet Allocation (LDA) model on Facebook Page Likes data [20,21]. First, for each user/year combination a document was created collapsing all Facebook Page Likes as expressed by its category. In this context, a document consists of a list of Pages Likes categories, as many as the Likes expressed by the user. This step results in 6100 documents (610 users × 10 time points). To identify the optimal number of topics, we trained a set of competing LDA models with the following k numbers of topics: 5, 10, 15, 20, 25, 30, 35, 40, 45, 50, and 55. Model training was performed on a random split including 90 % of the sample, while 10 % were used for model validation. The performance of the competing LDA models was compared by computing the perplexity statistic on the validation set (Wallach et al., 2009); the optimal number of topics was selected using the heuristic approach proposed by Zhao and colleagues [32], which is based on examination of the rate of perplexity change (RPC) across LDA models. The coherence of LDA-derived topic-words association was also examined visually using word clouds. Eventually, based on the RPC heuristic procedure and semantic coherence (as assessed by inspecting top words for each topic, i.e., in this context, page categories more strongly related to the specific topic), we selected k = 20 as the optimal number of topics for this dataset. As a last analytical step, the selected model was applied to all available documents to generate the topic proportion scores. LDA analyses were performed using the Mallet software, version 2.08 [33]. For each topic, the score consisted of the number of words (i.e., Page Likes) assigned to a particular topic. Top words and descriptive statistics for LDA topics emerging from Facebook Page Likes are reported in full in the Supplementary Materials.

Next, we aimed to analyze the association between topics emerging from participants’ Facebook Page Likes, and personality traits. For this purpose, we computed Spearman correlation coefficients that quantified the association between personality scores and the topic scores across a timeline extending up to 10 years prior to the Big Five personality assessment. Additionally, we also computed partial Spearman correlations controlling for differences in gender, age, and education level. This analytical step was performed to isolate the unique contribution of personality traits to topic preferences beyond the variance explained by these demographic variables. This approach acknowledges these demographic variables as crucial covariates that can influence both personality traits and interests, potentially confounding their association [34]. For each trait and topic combination, the average correlation over ten data points was calculated. Bootstrap resampling (1000 samples) was employed to estimate the 95 % confidence intervals for these averages.

Next, we investigated the stability in time of associations between personality and topics emerging from Facebook page Likes. To this aim, Spearman correlation coefficients at different time points (t0-t9) were compared with the Fisher's Z transformation procedure [35]. Because of the large number of pairwise comparisons per trait-topic combination (i.e., all possible comparison of 10 timepoints, resulting in 45 pairwise comparisons), when testing differences between correlations, Bonferroni correction was applied to the nominal level of significance (α = 0.05). Analyses were conducted using SPSS version 23 [36], and the statsmodels [37] and NumPy [38] Python packages.

## 3 Results

**Fig. 1:**
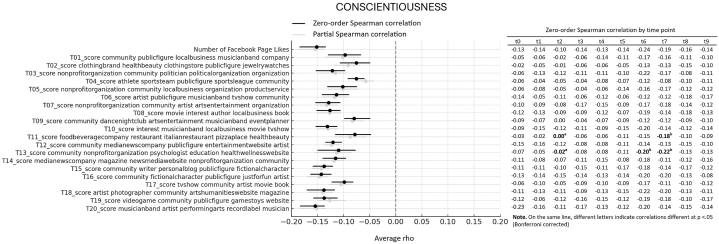
Forest plot of average Spearman correlations (and 95 % C.I.) between Conscientiousness and page Likes variables.

**Fig. 2:**
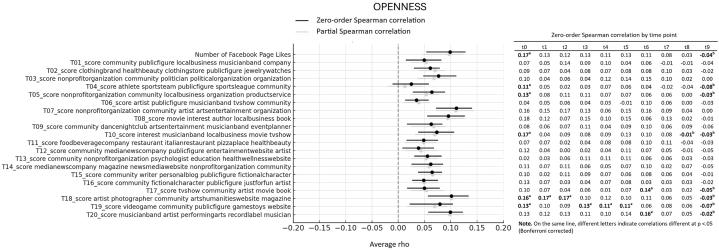
Forest plot of average Spearman correlations (and 95 % C.I.) between Openness to Experience and page Likes variables.

**Fig. 3:**
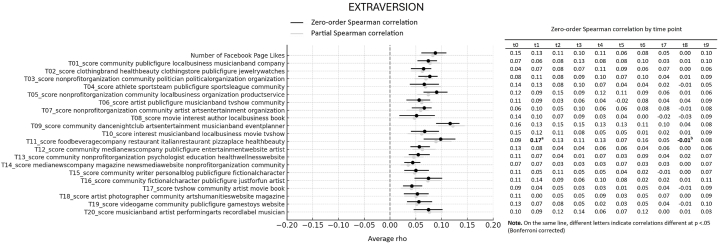
Forest plot of average Spearman correlations (and 95 % C.I.) between Extraversion and page Likes variables.

**Fig. 4:**
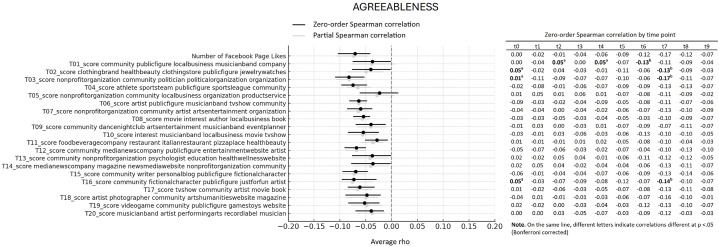
Forest plot of average Spearman correlations (and 95 % C.I.) between Agreeableness and page Likes variables.

**Fig. 5:**
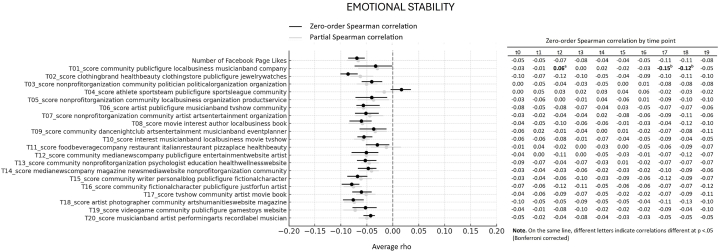
Forest plot of average Spearman correlations (and 95 % C.I.) between Emotional Stability and page Likes variables.

First, we looked at the average association between Big Five personality traits and interests showed through Likes expressed on Facebook Pages over 10 years of online activity. More specifically, we computed the average of Spearman correlations emerging from 10 datapoints (one for each year prior to the assessment), reflecting online activity over 10 years before the personality assessment. All average correlations and relative bootstrap 95 % confidence intervals are visualized in the forest plots presented Fig. 1, Fig. 2, Fig. 3, Fig. 4, Fig. 5. Note that in the figures, t0 indicates the same year of personality assessment, and the t1-t9 labels indicate 1–9 years prior to the original personality assessment (t0). Here we only comment on those topics for which we found an average correlation ≥ |0.10| (i.e., a non-negligible correlation according to Cohen's guidelines for effect size [39]).

For Conscientiousness (Fig. 1), there were consistent negative correlations across various interests (i.e. 16 out 20 topics showed significant negative correlations), with the most pronounced being a negative correlation with Likes on pages by musician bands, artists, and related entertainment fields (topic 20, average *ρ* = −0.15, 95 % CI [−0.18, −0.14]). This trend of negative correlations extended to Facebook pages related to video games and games/toys (topic 19, average ρ = −0.14, 95 % CI [−0.16, −0.11]), pages by artists and photographers (topic 18, average *ρ* = −0.14, 95 % CI [−0.17, −0.12]), writers and personal blog pages (topic 15, average *ρ* = −0.14, 95 % CI [−0.16, −0.12]), and fictional characters and “just for fun” pages (e.g., pages or profiles that are created primarily for entertainment, humor, or to share lighthearted content) (topic 16, average *ρ* = −0.14, 95 % CI [−0.16, −0.12]). Notably, interests in non-profit organizations and political organizations also showed significant negative correlations (topic 3, average *ρ* = −0.12, 95 % CI [−0.15, −0.10]). Correlations with remaining topics were all negative.

Openness to Experience showed relevant positive correlation with 4 out 20 topics (Fig. 2). In particular, the trait correlated with page related to artistic and cultural fields, including pages of non-profit organizations, performance, theaters, musicians and bands, magazines, and festivals (topic 7, average *ρ* = 0.11, 95 % CI [0.07, 0.14]) and a general interest in media and entertainment, including movies and books (topic 8, average *ρ* = 0.10, 95 % CI [0.06, 0.13]). Similar positive associations were observed with pages of artists and photographers (topic 18, average *ρ* = 0.10, 95 % CI [0.06, 0.13]), as well as pages related to musician and bands, artists, and related entertainment fields (topic 20, average *ρ* = 0.10, 95 % CI [0.06, 0.12]). Finally, Extraversion (Fig. 3) displayed non-negligible average positive correlations with 2 out 20 topics, and specifically with page relates to dance nightclubs, arts, and entertainment (topic 9, average *ρ* = 0.12. 95 % CI [0.09, 0.13]), as well as with food and beverage companies and restaurants (topic 11, average *ρ* = 0.10. 95 % CI [0.07, 0.13]). Agreeableness (Fig. 4) and Emotional Stability (Fig. 5) did not reveal relevant average associations with preference expressed through Facebook Pages over the considered 10-year period of online activity.

Now that when looking at the overall number of Likes to Facebook Pages, there was a negative correlation between the number of Likes and Conscientiousness. This suggests that individuals that are more conscientious are likely to express fewer Page Likes. The relationship was relatively stronger compared to the other traits and indicated a consistently negative association over time (average *ρ* = −0.15, 95 % CI [−0.18, −0.13]. There was also small positive correlation between the number of Page Likes and Openness to Experience (average *ρ* = 0.10, 95 % CI [0.05, 0.13]. This suggests that individuals who are more open to new experiences tend to produce a higher number of Likes, regardless of the topic of interest.

Still regarding the average correlations between personality and topics emerging from Facebook Page Likes, note that Fig. 1, Fig. 2, Fig. 3, Fig. 4, Fig. 5 also show partial Spearman correlations computed while controlling for demographic variables. Upon comparing zero-order and partial Spearman correlations a remarkable overlap was observed (Fig. 1, Fig. 2, Fig. 3, Fig. 4, Fig. 5). This overlap indicates that demographic variables, which were controlled for in the partial correlations, do not appear to significantly confound the associations between personality traits and online preferences. This finding was consistent across the various topics of interest that exhibited both negligible and substantial correlations (i.e., average correlations ≥ |0.10|), suggesting that the intrinsic link between personality and online behavior stands robust against the potential confounding introduced by demographic differences. Note that all average zero-order and partial Spearman correlations and 95 % confidence interval are reported in full in Table S1, in the supplementary materials. Additionally, zero-order Spearman correlations (and relative statistical significance) computed between personality and Facebook Likes variables for all the ten datapoints are also reported in the supplementary materials, Table S2.

Finally, the analysis involved investigating differences in Spearman correlation coefficients between the Big Five personality traits and interests as expressed through Facebook Page Likes at different time points. For all traits × topic combinations, point estimate of correlations at different time points are reported in Fig. 1, Fig. 2, Fig. 3, Fig. 4, Fig. 5; results of pairwise comparisons are reported by marking correlations that differ significantly (p < 0.05, Bonferroni corrected) at different time points with distinct letters. Overall, based on the number of topics showing significant variations in correlation with the traits over time, these associations demonstrated remarkable stability. Based on the number of comparisons showing evidence of variations, the Openness to Experience trait exhibited less stability in these associations (By descending number of significant pairwise comparisons: Openness: n = 14; Agreeableness: n = 5; Conscientiousness: n = 3; Emotional Stability: n = 2; Extraversion: n = 1). Indeed, for the Openness trait, seven topics (i.e., topics 4, 5, 10, 17, 18, 19, and 20) and the number of Facebook Page Likes showed stronger correlations with the trait when computed at more recent time points (t0 to t5, depending on the topic) compared with time points further from the personality assessment (t8, t9). This suggests that the Openness trait tends to show a stronger association with preferences for online content depending on their recency. For transparency, the results of testing for significance of pairwise comparisons (i.e., uncorrected p values) are reported in full in Table S3 in the supplementary materials.

## 4 Discussion

The present study explored two hypotheses on the association between brief measures of Big Five personality traits and online behaviors in Facebook users, specifically the expression of interests through Facebook page Likes. The first hypothesis posited that specific associations would emerge between Big Five personality traits and preferences emerging from Facebook page Likes. Overall, results largely supported this hypothesis, in that observed correlations between personality traits and Facebook Page Likes aligned with existing literature on personality-behavior associations. The negative correlation between Conscientiousness and various leisurely or entertainment interests reflects the trait's orientation towards structure, reliability, and practicality [40]. Conscientious individuals often prioritize productivity and organization, which may explain their lower inclination towards such interests. In turn, we found positive correlations between Openness to Experience and a variety of artistic and cultural interests; these align with the trait's association with creativity, curiosity, and appreciation for diverse cultural expressions [41,42]. The positive correlations with Extraversion, particularly with social and lively entertainment options, are consistent with the trait's sociability and need for external stimulation [43]. It should be noted, however, that these correlations were generally small; additionally two of the Big Five personality traits, namely Agreeableness and Emotional Stability, did not show relevant average associations with the preferences expressed through Facebook Pages over the considered 10-year period. The lack of significant average correlations for these traits is an interesting point and aligns in part with findings from meta-analyses highlighting a weaker association between digital traces of social media activity and both Agreeableness and Neuroticism (the inverse of Emotional Stability) when compared with other traits (i.e., Conscientiousness, Extraversion, and Openness to Experience) [44]; for a study focusing on Facebook, see Ref. [45]). Note however that our results on Conscientiousness diverge significantly from those by Kosinski and colleagues [13] in showing this trait has the one most consistently connected with topics emerging from Facebook Likes. The observed inconsistency in the results merits further exploration; it may be related to differences in the research approach, the methods used to evaluate personality, and the distinct characteristics of the study participants, such as their number, gender, and age distribution.

The second hypothesis in our study proposed that the associations emerging between personality traits and preferences for specific online content would prove stable in over the considered time frame. Results mostly supported this hypothesis, as correlations between personality scores and individual difference in topics emerging from Facebook Page Likes showed little variation in size over the course of the previous decade of data points. The stability of these correlations over time suggests a consistency in how personality traits relate to interests and preferences. Moreover, although indirectly, this aligns with the understanding that the Big Five traits are relatively stable over time [6,9]. Of note, the Openness to Experience trait showed less stability than the other traits in the association with specific topics, including preferences for arts and media, such as artists and photographers (topic 18), entertainment media including tv shows, movies, and books (topic 17), videogames (topic 19), musicians and bands (topic 10, topic 20). Overall, when inspecting the patterns emerging from the correlations, results suggested that the Openness trait might show stronger associations with recent interests as opposed to old ones. Although partially conflicting with our hypothesis of stability of trait-topic associations, the significant variations in strength of associations with Openness across older and newer interests can be understood in light the characteristics of the trait, which includes a constant striving for new and diverse interests (i.e., a need *for psychological interest diversity*; on this, see Ref. [46] for a recent study investigating on large datasets of behavioral data, including Facebook page Likes data).

The extended timeframe employed in the present study (i.e., online behaviors were measured over a ten-year span) is a significant strength, providing robust evidence of how even a short personality assessment relates consistently with behaviors over a long period of time. Additionally, the use objective recordings of online behaviors enhance the study's empirical validity and limits the influence of common-method bias on emerging associations. Despite these strengths, this study has limitations that affect its generalizability. The use of a brief Big Five personality assessment, and a relatively small and skewed sample in terms of gender distribution, may have not fully captured the complexities of the traits or represent the broader population. In addition, the reliance on Facebook Page Likes as a proxy for online behavior could introduce a form of selection bias, as it does not capture the entirety of an individual's online activities. The landscape of social media is vast and varied, and users may express their interests through other platforms and behaviors not captured by Facebook Page Likes. Furthermore, technological changes over the ten-year span of the data examined in this study could have influenced the nature of Facebook usage and the significance of Page Likes as a form of online behavior. The introduction of new features, changes in privacy settings, and shifts in user engagement patterns may alter how individuals interact with the platform and express their online preferences, potentially affecting the long-term validity of our findings.

Lastly, while our study provides valuable insights into the relationship between personality traits and digital footprints on Facebook, it is essential to recognize the limitation of generalizing these findings to other digital platforms. The digital expression of personality is a multifaceted phenomenon that can manifest differently across various online contexts.

## 5 Conclusions

To conclude, the results of this study support the notion that online behaviors, such as Facebook Page Likes, may serve as long-term indicators of individual differences in the Big Five personality traits. These findings align with previous research, which has demonstrated that digital traces of online activity can be used to infer individual differences in the Big Five personality traits. Notably, our results extend beyond the existing literature by showing that even years-old recordings of online behavior may retain some predictive power over users' current personality scores. It should be noted, however, that the observed associations were generally small and varied depending on the specific personality trait; Conscientiousness, Openness, and, to a lesser extent, Extraversion, exhibited stronger correlations with online preferences compared to other traits. Additionally, the overall stability of these associations over time was notable, though Openness to Experience showed stronger associations with more recent interests, suggesting a dynamic aspect to how this trait interacts with online behaviors. Our findings underscore the potential of digital footprints for personality assessment and highlight the need for future studies to explore the strength and stability of these associations over time across various online platforms and types of digital traces (e.g., texts, images) and personality traits.

## Ethics statement

This study was reviewed and approved by the ethics committee of the University of Turin, Italy, with the approval number: n° 88721.

## Data availability statement

Data cannot be shared publicly because it includes confidential data. Data will be made available on request.

## CRediT authorship contribution statement

**Davide Marengo:** Writing – review & editing, Writing – original draft, Visualization, Validation, Methodology, Investigation, Formal analysis, Data curation, Conceptualization. **Michele Settanni:** Writing – review & editing, Validation, Supervision, Methodology, Funding acquisition, Data curation, Conceptualization.

## Declaration of AI and AI-assisted technologies in the writing process

During the preparation of this work, the author(s) used Openai's ChatGPT 4 in order to check grammar and spelling, and improve overall readability of the text. After using this tool/service, the author(s) reviewed and edited the content as needed and take(s) full responsibility for the content of the publication.

## Declaration of competing interest

The authors declare that they have no known competing financial interests or personal relationships that could have appeared to influence the work reported in this paper.
